# Pits in Metals Caused by Collision With Liquid Drops and Rigid Steel Spheres

**DOI:** 10.6028/jres.064A.006

**Published:** 1960-02-01

**Authors:** Olive G. Engel

## Abstract

A pit-depth-versus-velocity equation developed earlier was tested further with experimental data obtained using target plates of electrolytic tough pitch copper, 1100–O aluminum, and 2024–O aluminum, the static strength properties of which were measured by testing tensile specimens. The projectiles used to produce the pits were mercury drops, waterdrops, and steel spheres. It was found that the numerical constants in the equation for projectiles that flow during and as a result of the collision are different from those for projectiles that do not flow (hardened steel spheres). Curves calculated using the equation were found to be in acceptable agreement with experimental pit-depth-versus-velocity data for collisions of the indicated projectiles with target plates of the three metals used with the exception of the case of steel-sphere impingement against 2024–O aluminum alloy. In this case work-hardening of the target metal seems to foster a mode of pit formation that was not considered in the development of the pit-depth-versus-velocity equation.

## 1. Introduction

Collisions between liquids and solids in all of the possible projectile-target combinations in which they can occur have been topics for research. Some of these are: solid-against-solid collisions (artillery experiments), solid-against-liquid collisions (water-entry problems), liquid-against-solid collisions (high-speed rain-erosion research), and liquid-against-liquid collisions (impact of solids at meteor velocities). Work has been done at the National Bureau of Standards toward developing an equation that will give pit depth as a function of impingement velocity for collisions of target plates of the soft and medium hard metals with drops of liquids [1].^1^

The model on which the equation is based is the movement of the core of metal of the target plate immediately under the collision area with respect to the remainder of the plate. In order that the core of metal through the target plate under the collision area may be free to move, the side of the plate opposite to that on which the collision occurs must be a free surface. In addition to this condition on the target plate, it must not be so thin that it bends as a whole under the collision, or so thick that the spread of the compressional wave that passes through it as a result of the collision is appreciable.

In the development of this pit-depth-versus-velocity equation, such characteristic fluid-flow parameters as the Weber number and the Reynolds number were neglected. The equation should, therefore, apply equally well to pits caused by collisions of solid spheres with target plates of the soft and medium hard metals.

In the case of collisions of solid-sphere projectiles that flow like a liquid drop during and as a result of the collision, the equation should apply without modification even of the numerical constants. It has been found that the equation does produce curves that fit pit-depth-versus-velocity data for high-speed collisions of soft ductile metal spheres against targets of the same metals [1].

For the case of collisions of hardened steel spheres, the numerical constants in the equation will be different. These spheres do not flow during the collision; none of the collision energy is lost in the flow of the projectile and, therefore, a larger amount of it is used in forming the pit.

Pit-depth-versus-velocity data^2^ for high-speed collision of liquid drops with metal plates were used to evaluate the numerical constants in the equation and to test the equation. These data were of a preliminary nature. The yield strength and the speed of sound of the metals used for the target plates were not determined by experiment. The speed of sound in this case is the speed of irrotational waves in an infinite medium.

It is important to know the static yield strength of the target metal. Although it is the dynamic yield strength that must be used in the equation, in most cases the dynamic yield strength may be expected to vary in the same direction that the static yield strength varies for different heat-treatment states of a given metal. Different sets of pit-depth-versus-velocity data will not, in general, be comparable unless the static yield strength of the metal target plates that are used is essentially the same. Furthermore, the static yield strength can be used to calculate the dynamic yield strength in the case of the duralumins [2] and closely related aluminum alloys.

The work described in this paper is an effort to test the equation further by determining the depth of pits that result from impingement of waterdrops, mercury drops, and rigid steel spheres against target plates of 1100–O aluminum, 2024–O aluminum, and annealed electrolytic tough pitch copper, the static yield strengths of which have been determined by experiment. The work described was conducted under the sponsorship of the Materials Laboratory, Directorate of Laboratories, Wright Air Development Center. The experimental work was done at Convair, Division of General Dynamics Corp., in San Diego, Calif., and at the U.S. Naval Research Laboratory in Washington, D.C.

## 2. Materials

### 2.1. Preparation of the Target Plates

Plates of the metals were obtained in 2.5-cm (1-in.) thickness so that 15.2- by -15.2-cm (6- by -6-in.) target plates cut from them for use in experiments involving impingement of 0.5556-cm- ( 732-in.-), 0.7938-cm- ( 516-in.-), and 1.270-cm- (½-in.-) diam steel spheres, would be approximately 2 to 4.5 sphere diameters thick. Tensile specimens for determining the static yield strength and small 0.317-cm- (⅛-in.-) thick target plates for use in experiments involving collision with 0.1-cm and 0.2-cm drops of mercury and water were machined from some of this material.

The 1100–O aluminum was obtained from the Davenport Works of the Aluminum Co. of America in Riverdale, Iowa. The material was furnished in mill finish and was as scratch-free as was commercially feasible. For the annealing process the objects were placed in the furnace while it was cold. The furnace temperature was then brought up to 343° C (650° F) and was held at 343° C (650° F) for 4.5 hr in the case of the large target plates and the tensile specimens and for about 2 hr in the case of the small target plates. The furnace was then turned off and the objects were allowed to cool with the furnace. The tensile specimens were suspended in the vertical position during the annealing process to prevent sagging.

The 2024–O aluminum was obtained as 2024–T4 aluminum at the U.S. Naval Research Laboratory in Washington, D.C. For the annealing process the tensile specimens and the target plates of this aluminum alloy were put into the furnace after it had been raised to a temperature of 399° C (750° F). They were heated at 399° C (750° F) for 4 hr, cooled to 166° C (350° F) at a rate of 25.5° C (46° F) per hour, and then removed from the furnace.

The electrolytic tough pitch copper was obtained from the American Brass Co. For annealing, the objects were placed in the furnace after it had attained a temperature of 427° C (800° F). The large target plates were kept in the hot furnace for approximately 1 hr, the small target plates for about 40 min, and the tensile specimens for about 30 min. The objects were in each case removed from the furnace at the end of the specified time and were air cooled. The copper oxide that formed was removed from the target plates by pickling in dilute acid and by gentle abrasion.

### 2.2. Static Tensile Properties of the Metals

The tensile specimens were standard ASTM test specimens having 1.283-cm (0.505-in.) diameter in accordance with ASTM designation E 8–54 T. They were tested in a standard testing machine in the NBS Engineering Mechanics Section using autographic recording equipment. The data obtained were yield strength (0.2 percent offset), tensile strength, and elongation in 5.1 cm (2 in.). The test results are given in table 1.

## 3. Liquid-Against-Solid Collisions

The small target plates of 1100–O aluminum, 2024–O aluminum, and annealed electrolytic tough pitch copper were sent to Convair, Division of General Dynamics Corp., in San Diego, Calif., to be fired into drops of mercury (0.1 cm and 0.2 cm in diam) and drops of water (0.2 cm in diam) at high speed. When the tests were made, the 1100–O aluminum targets were not fired because they were too thin.

The mercury drops used in the firings were individually weighed on an analytical balance, and the diameters of the drops were calculated. The actual diameters of the mercury drops and waterdrops varied in most cases by less than ±10 percent of the nominal size. The depths of the pits produced were measured at Convair using an optical micrometer; the depth measurements were reported to be good to ±0.0013 cm (±0.0005 in.).

The theoretical curves for the collisions of liquid drops against solid targets were obtained using the equations [1]

$$\delta^{\prime }=\frac{7.2dz}{c\left(z+z^{\prime }\right)}\left[V-V_{i}\right]$$ (1)

and

$$V_{i}=\frac{19E^{\prime }\left(z+z^{\prime }\right)}{{\left(\rho c^{\prime }z^{\prime 3}\right)}^{\frac{1}{2}}}$$ (2)

in which *δ′* is pit depth, *d* is drop diameter, *c* is the speed of sound as defined previously, *ρ* is the density, *z* is the acoustic impedance (*z=cρ*), *E*′ is the dynamic compressive yield strength of the target metal, *V_i_* is the smallest impingement velocity at which a permanent pit is made, and *V* is the impingement velocity. Primed quantities refer to the material of the target plate and unprimed quantities refer to the material of the liquid drop. All quantities are in cgs units. Values of these quantities for the materials used for projectiles and targets are listed in table 2.

The development of eqs (1) and (2) has been given previously [1]. The conditions for valid use of these equations were discussed in section 1.

### 3.1. Collisions Between Metal Target Plates and Mercury Drops

**Figure 1:**
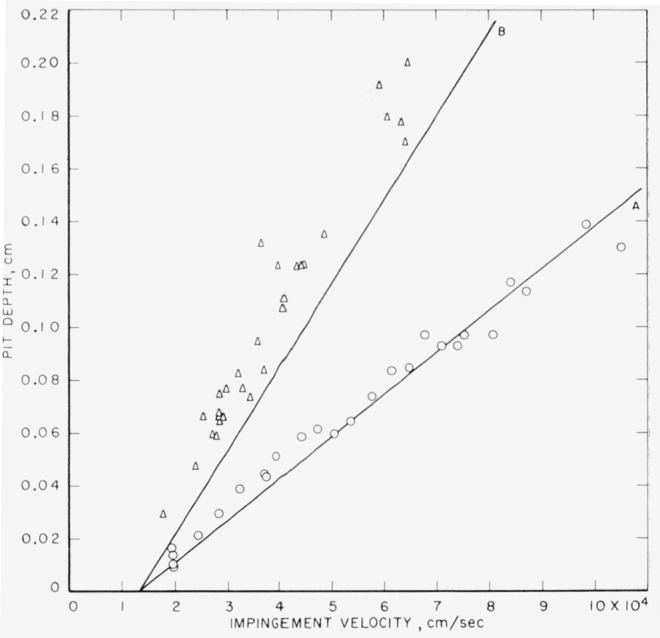
Collisions of mercury drops of two sizes against annealed electrolytic tough pitch copper. Curve A, calculated using eqs (1) and (2) for 0.1-cm drops; ⊙, observed depth produced by collision with 0.1-cm drops; Curve B, calculated using eqs (1) and (2) for 0.2-cm drops; ◬, observed depth produced by collision with 0 2-cm drops.

The experimental pit depths for collisions of 0.1- and 0.2-cm mercury drops against target plates of annealed electrolytic tough pitch copper are listed in table 3. They are plotted in figure 1 along with theoretical curves calculated using eqs (1) and (2) and the data in table 2. The theoretical curves are in relatively good agreement with the experimental points. The effect of the change in drop size is properly accounted for by the theoretical equations. Curve A, calculated for the 0.1-cm drop size, is in better agreement with the observed depths produced by collision with 0.1-cm drops than curve B, calculated for the 0.2-cm drop size, is with the observed depths produced by collision with 0.2-cm drops. The assumption of spherical drops used in calculating drop diameters from the weight of the mercury drops can be expected to be more accurate for the smaller drop size. In this connection, it is noteworthy that there is more scatter in the experimental data for the 0.2-cm drop size.

On the other hand, if the calculated intercept velocity, *V_i_*, had been a little smaller, the fit would have been better. The dynamic compressive yield strength reported by Whiffin [2] for electrolytic copper was used for *E*′ in computing *V_i_* for the theoretical curves (see table 2). It is not known if the electrolytic copper for which Whiffin [2] obtained the dynamic compressive yield strength was equivalent to the electrolytic tough pitch copper that was used for the target plates. Whiffin [2] did not report the static yield strength of the electrolytic copper that he used; he described it as being normally very soft and giving no definite indication of yield strength in static compression tests. No formula exists for copper by means of which the dynamic compressive yield strength can be calculated from the static yield strength; however, the latter provides a means of identification that can be reproduced by others.

**Figure 2:**
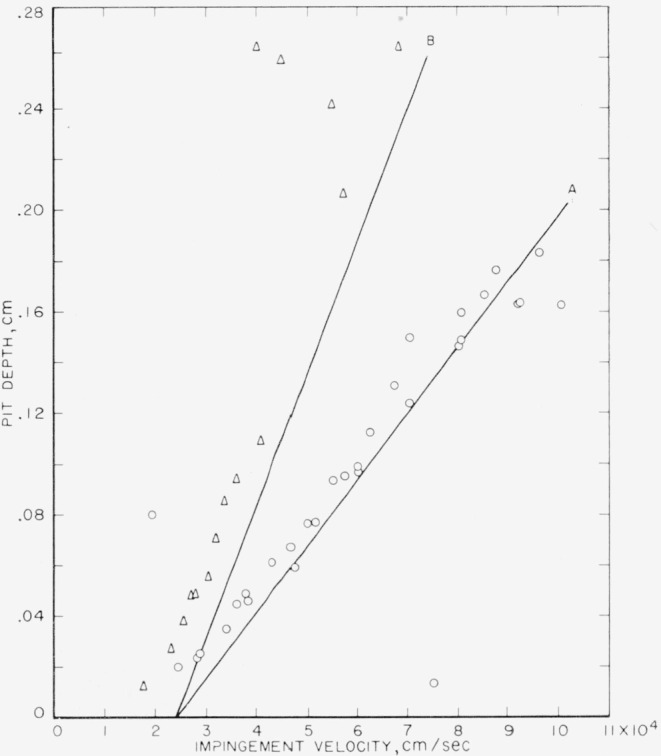
Collisions of mercury drops of two sizes against 2024–O aluminum Curve A, calculated using eqs (1) and (2) for 0.1-cm drops; ⊙, observed depth produced by collision with 0.1-cm drops; Curve B, calculated using eqs (1) and (2) for 0.2-cm drops; ◬, observed depth produced by collision with 0.2-cm drops.

The experimental pit depths for collisions of 0.1- and 0.2-cm mercury drops with target plates of 2024–O aluminum are listed in table 4. They are plotted in figure 2 with the theoretical curves calculated using eqs (1) and (2) and the data in table 2. The theoretical curves are in reasonably good agreement with the experimental points. As was found to be true in figure 1, the effect of the change in drop size is properly accounted for by the theoretical equations. As in the case of the copper targets, curve A, calculated for the 0.1-cm drop size, is in better agreement with the observed depths produced by collision with 0.1-cm drops than curve B, calculated for the 0.2-cm drop size, is with the observed depths produced by collision with 0.2-cm drops. There is quite a bit of scatter in the experimental points for both drop sizes.

As in the case of the pit-depth-versus-velocity data for copper, there would be better agreement between the theoretical curves and the empirical data if the calculated intercept velocity, *V_i_*, were smaller. In the case of the 2024–O aluminum, the dynamic yield strength used for *E*′ in computing *V_i_* was calculated from the measured static yield strength (see table 2) and should be fairly reliable.

The numerical constants used in eqs (1) and (2) were originally chosen [1] using pit-depth-versus-velocity data (see footnote 2) for metals whose static yield strength and whose sound speed in infinite medium were not measured experimentally. It may be found necessary to change the constants in the equations to some extent when more pit-depth-versus-velocity data become available using targets of metals for which these quantities have been measured.

Although eq (2) appears to be the most acceptable expression for the intercept velocity on the basis of the available experimental data for collisions of liquid drops against solids [1], it is possible that, when more data are obtained and the problem is studied further, it may be found necessary to modify it.

### 3.2. Collisions Between Metal Target Plates and Waterdrops

**Figure 3:**
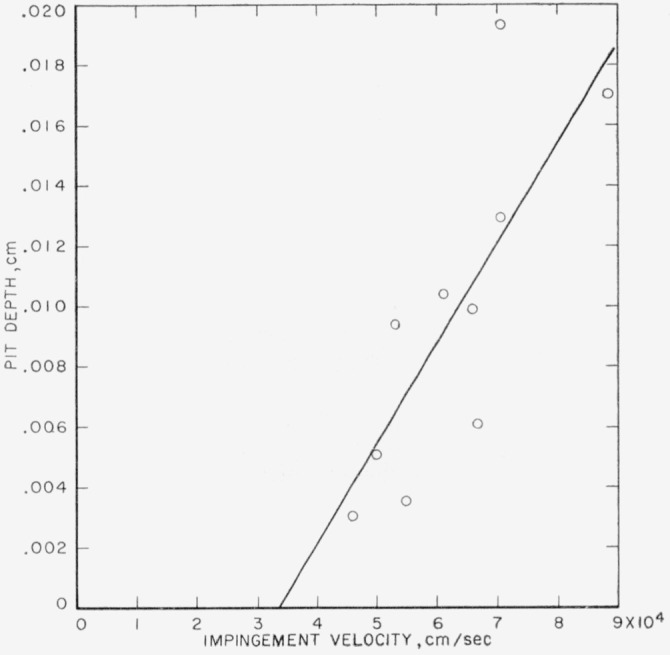
Collisions of 0.2-cm waterdrops against annealed electrolytic tough pitch copper ——, calculated using eqs (1) and (2) for 0.2-cm drops; ⊙, observed depth produced by collision with 0.2-cm drops.

The experimental pit depths for collisions of 0.2-cm waterdrops against target plates of annealed electrolytic tough pitch copper are listed in table 5. They are plotted in figure 3 with the theoretical curve calculated using eq (1) and (2) and the data in table 2. The theoretical curve is in acceptable agreement both as to slope and intercept with the experimental data. There is, unfortunately, a large amount of scatter in the experimental data which reduces their effectiveness as a check of the theoretical equations.

**Figure 4:**
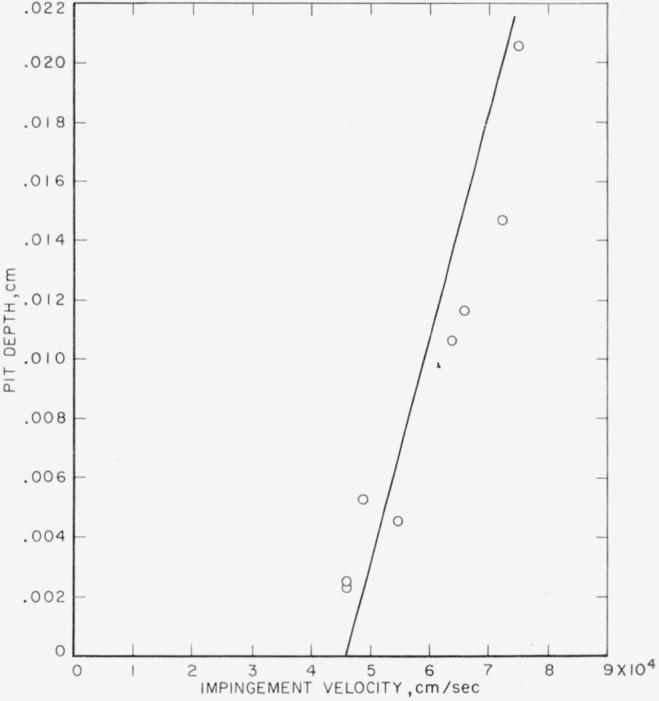
Collisions of 0.2-cm waterdrops against 2024–O aluminum. ———, calculated using eqs (1) and (2) for 0.2-cm drops; ⊙, observed depth produced by collision with 0.2-cm drops.

The experimental pit depths for collisions of 0.2-cm waterdrops against target plates of 2024–O aluminum are listed in table 6. They are plotted in figure 4 with the theoretical curve calculated using eqs (1) and (2) and the data of table 2. The theoretical curve is a good fit for the experimental data both as to slope and intercept. This is more significant than the extent of agreement found between the theoretical curve and the experimental data in figure 3 because there is considerably less scatter in these data than in those obtained with the copper targets.

The agreement found between the theoretical curves and the experimental data in figures 3 and 4 is an indication that eqs (1) and (2) may be reliable in their present form for calculating the depths of pits formed in the soft and medium hard metals as a result of collision with liquid drops. Although the speed of sound in mercury is nearly identical with the speed of sound in water, the density, and, therefore, the acoustic impedance, of mercury is very much higher than that of water. Further test of the equations should be made, however, using drops of a liquid that has a sound speed different from that of mercury or water.

## 4. Solid-Against-Solid Collisions

Because the pit-depth-versus-velocity equation developed [1] for collisions of target plates of the soft and medium hard metals with liquid drops ignored such characteristic fluid-flow parameters as the Weber number and the Reynolds number, it should apply equally well to pits produced by collision of solid spheres with target plates of the same types of metals. The equation may, in fact, be substantiated further with data of this kind. To explore this possibility, 2.5-cm- (1-in.-) thick plates of 1100–O aluminum, 2024–O aluminum, and annealed electrolytic tough pitch copper were used as targets for steel sphere impingement. The test firings were made at the U.S. Naval Research Laboratory in Washington, D.C., under the direction of Mr. Wilfred J. Ferguson and Mr. Harry O. Ewing. Most of the shots were made using a target mounting in which the plate was given edge support only. In this form of mounting the rear face of the target plate was a free surface. These data are presented and discussed in sections 4.1, 4.2, and 4.3. One set of data was obtained for collisions of steel spheres against 2024–O aluminum target plates backed with a 12.7-by-15.2-cm (5-by-6-in.) steel supporting block 7.6-cm (3-in.) thick. In this form of mounting the rear face of the target plate was not a free surface. These data are presented and discussed in section 4.4.

The velocity measurements were made using a Potter chronograph. The base length was 30.5 cm (12 in.). The chronograph was started and stopped by breaking conducting grids. The distance from the midpoint of the base length to the target face was 58.4 cm (23 in.); the velocity measurements were not corrected for deceleration of the steel spheres during transit over this distance.

The steel spheres were SKF Grade 1 and had an approximate hardness of 60 on the Rockwell C scale. These spheres have a high order of accuracy in dimensions because they are made for ball bearings. Three sphere sizes were used for the firings: 0.5556-cm ( 732-in.), 0.7938-cm ( 516-in.), and 1.270-cm (½-in.) diameter.

The depths of the pits produced by impingement of the steel spheres against target plates of the three metals were measured using an Ames dial gauge graduated in mils. Each depth measurement is the difference between the dial reading at the pit bottom and an average of four dial readings taken on the surface of the target plate around the mouth of the crater.

### 4.1. Collisions of Steel Spheres With Target Plates of 1100–O Aluminum

The velocities at which the shots were made and the depths of the pits produced in the 1100–O aluminum plates are given in table 7 for the three sizes of steel spheres used.

The pit-depth-versus-velocity curve for collisions of steel spheres with 1100–O aluminum was first calculated according to the equations that were developed for collisions between metal target plates and liquid drops. Although the experimental pit-depth-versus-velocity data were found to lie along straight lines, the slope of the lines was found to be much steeper than that of the lines given by the pit-depth-versus-velocity equation for collision of liquid drops against target plates of the soft and medium hard metals. This is to be expected because in the case of the very hard steel-sphere projectiles most of the collision energy is used in forming the pits, whereas in the case of projectiles that flow during and as a result of the collision, part of the collision energy is used in the flow of the projectile.

To fit the experimental data, it was found by trial that it was necessary to increase the numerical constant in the expression for pit depth to 17.5 and to reduce the numerical constant in the expression for the intercept velocity to 1. With these changes, the pit-depth-versus-velocity equation for collisions of projectiles that neither flow nor undergo appreciable permanent yield with targets of the soft and medium hard metals is

$$\delta^{\prime }=\left[17.5dz/c\left(z^{\prime }+z\right)\right]\cdot \left[V-V_{i}\right]$$ (3)

where

$$V_{i}=E^{\prime }\left(z+z^{\prime }\right)/\left(\rho c^{\prime }z^{\prime 3}\right)\frac{1}{2}$$ (4)

**Figure 5:**
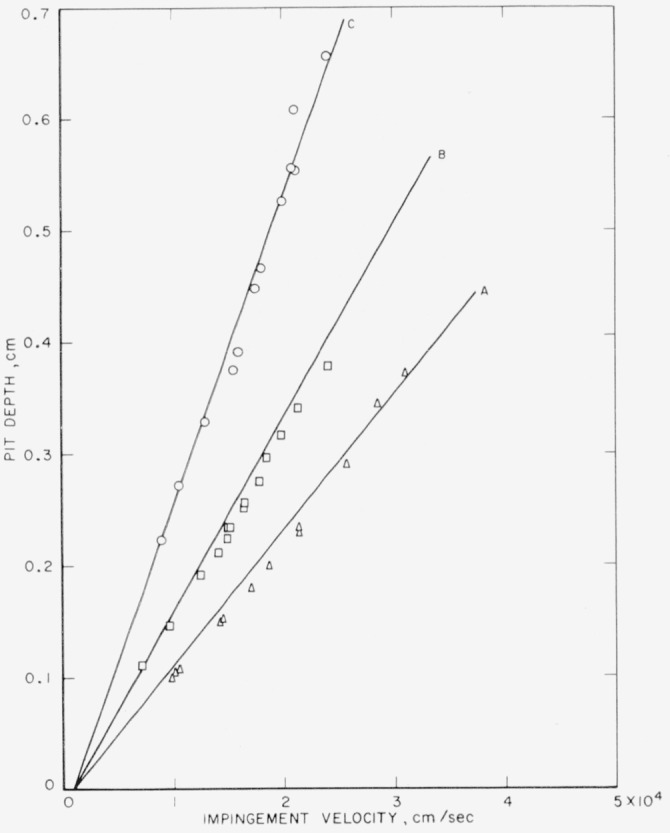
Collisions of steel spheres of three sizes against 1100–O aluminum. Curve A, calculated using eqs (3) and (4) for 0.5556-cm ( 732-in.) spheres; ◬, observed depth produced by collision with 0.5556-cm ( 732-in.) spheres; Curve B, calculated using eqs (3) and (4) for 0.7938-cm ( 516-in.) spheres; ⊡, observed depth produced by collision with 0.7938-cm ( 516-in.) spheres; Curve C, calculated using eqs (3) and (4) for 1.270-cm (½-in.) spheres; ⊙, observed depth produced by collision with 1.270-cm (½-in.) spheres.

The theoretical pit-depth-versus-velocity curves, calculated using eqs (3) and (4) and the data in table 2, for collisions of steel spheres of three sizes against 1100–O aluminum target plates are shown with the experimentally determined points in figure 5. The experimental points lie along the theoretical curves. In particular, it can be seen that the effect of a change in sphere size is properly accounted for by the equations.

### 4.2. Collisions of Steel Spheres With Target Plates of Annealed Electrolytic Tough Pitch Copper

**Figure 6:**
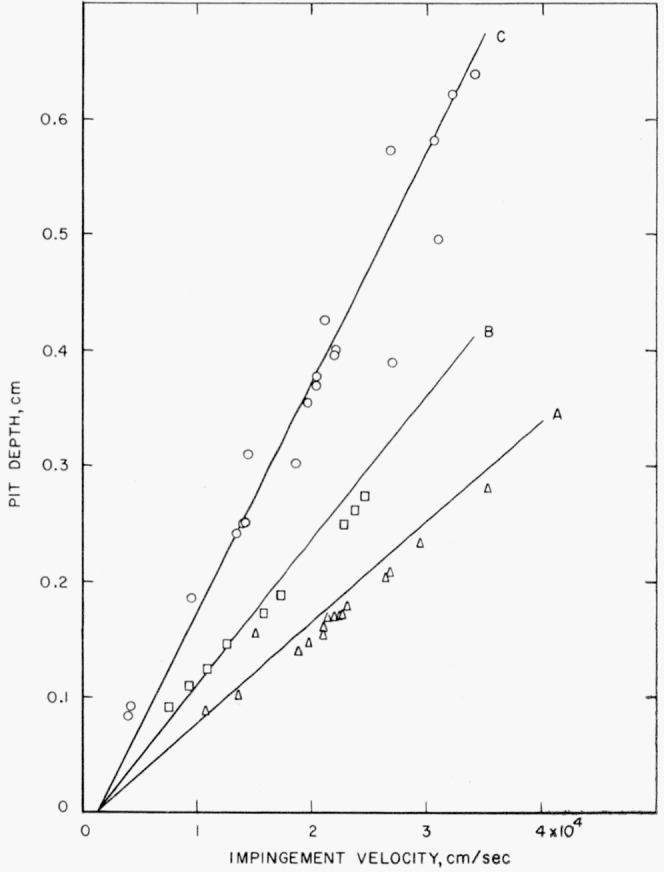
Collisions of steel spheres of three sizes against annealed electrolytic tough pitch copper. Curve A, calculated using eqs (3) and (4) for 0.5556-cm ( 732-in.) spheres; ◬, observed depth produced by collision with 0.5556-cm ( 732-in.) spheres; Curve B, calculated using eqs (3) and (4) for 0.7938-cm ( 516-in.) spheres; ⊡, observed depth produced by collision with 0.7938-cm ( 516-in.) spheres; Curve C, calculated using eqs (3) and (4) for 1.270-cm (½-in.) spheres; ⊙, observed depth produced by collision with 1.270-cm (½-in.) spheres

The velocity at which the shots were made and the depth of the pits produced with three sizes of steel spheres against 2.5-cm-(1-in.-) thick plates of annealed electrolytic tough pitch copper are given in table 8. Theoretical pit-depth-versus-velocity curves, calculated using eq (3) and (4) and the data in table 2, are shown with the experimentally determined points in figure 6. There is quite a bit of scatter in the experimental data for the 1.270-cm-(½-in.-) diam steel spheres. Nevertheless, there is, in general, good agreement between the theoretical curves and the observed points. The effect of a change in sphere size is properly accounted for by the equations.

### 4.3. Collisions of Steel Spheres With Target Plates of 2024–O Aluminum

**Figure 7:**
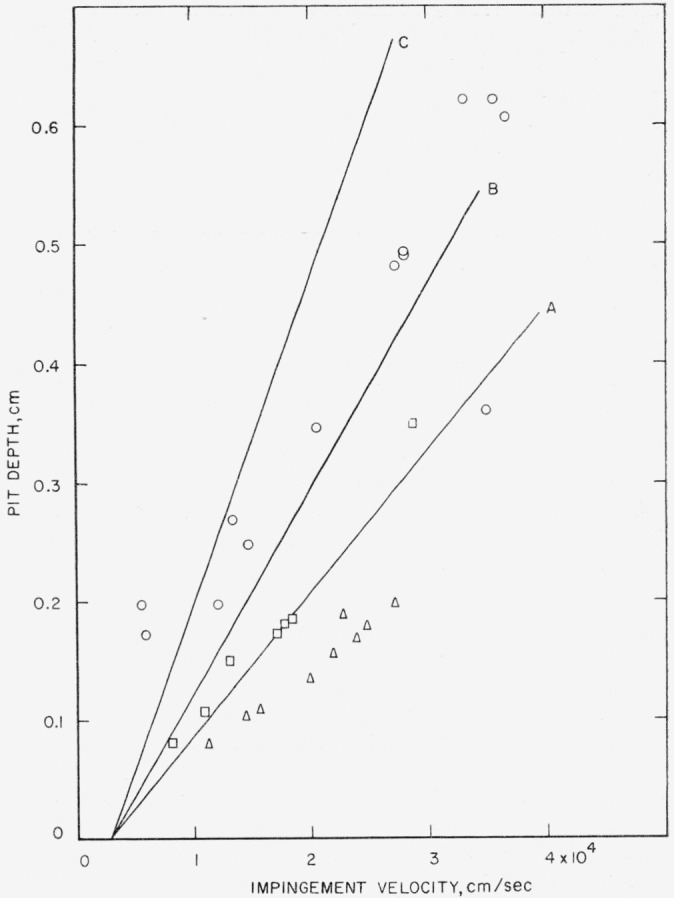
Collisions of steel spheres of three sizes against 2024–O aluminum. Curve A, calculated using eqs (3) and (4) for 0.5556-cm ( 732-in.) spheres; A, observed depth produced by collision with 0.5556-cm ( 732-in.) spheres; Curve B, calculated using eqs (3) and (4) for 0.7938-cm ( 516-in.) spheres; ⊡, observed depth produced by collision with 0.7938-cm ( 516-in.) spheres; Curve C, calculated using eqs (3) and (4) for 1.270-cm (½-in.) spheres; ⊙, observed depth produced by collision with 1.270-cm (½-in.) spheres.

The velocity at which shots with three sphere sizes were made against plates of 2024–O aluminum and the depths of the pits that were produced by the shots are given in table 9. Theoretical pit-depth-versus-velocity curves calculated using eq (3) and (4) and the data in table 2 are shown with the experimental data in figure 7. For each sphere size used, the experimental points lie below the theoretical curve.

Four possible reasons for this discrepancy were explored. (1) In steel-sphere collisions with materials as strong as 2024–O aluminum the steel sphere may be permanently deformed and in this way part of the collision energy may be diverted from pit formation, whereas in the case of the very soft metals all of the collision energy may go into pit formation. (2) In view of the fact that heat-treated 2024 aluminum is subject to spalling, energy may be diverted from pit formation into crack formation, although this is unlikely in the case of the annealed metal. (3) Sound is attenuated to different degrees in the target metals used. (4) The target metals that were used work-harden by different amounts.

To check the first possibility, two 1.270-cm-(½-in.-) diam steel spheres that were fired against target plates of annealed copper and 2024–O aluminum, respectively, were recovered. The sphere that struck the copper target plate had a collision velocity of 3.432×10^4^ cm/sec (1,126 ft/sec) and the sphere that struck the 2024–O aluminum target plate had a collision velocity of 3.650×10^4^ cm/sec (1,200 ft/sec). These spheres were examined with a microinterferometer in the NBS Engineering Metrology Section. It was found that the diameter measured through the impact area of the sphere shot into copper was 0.0025 cm (0.0010 in.) smaller than diameters measured outside of the impact area and that the diameter measured through the impact area of the sphere that was shot into 2024–O aluminum was 0.00020 cm (0.00008 in.) smaller than diameters measured outside of the impact area. On the basis of this evidence it cannot be concluded that a larger percentage of the impact energy is absorbed by a steel sphere on colliding with 2024–O aluminum than on colliding with annealed copper. In fact, the reverse is the case. Deformation of the steel sphere is not the cause of the divergence of the 2024–O aluminum experimental pit-depth-versus-velocity data from the theoretical curves.

**Figure 8:**
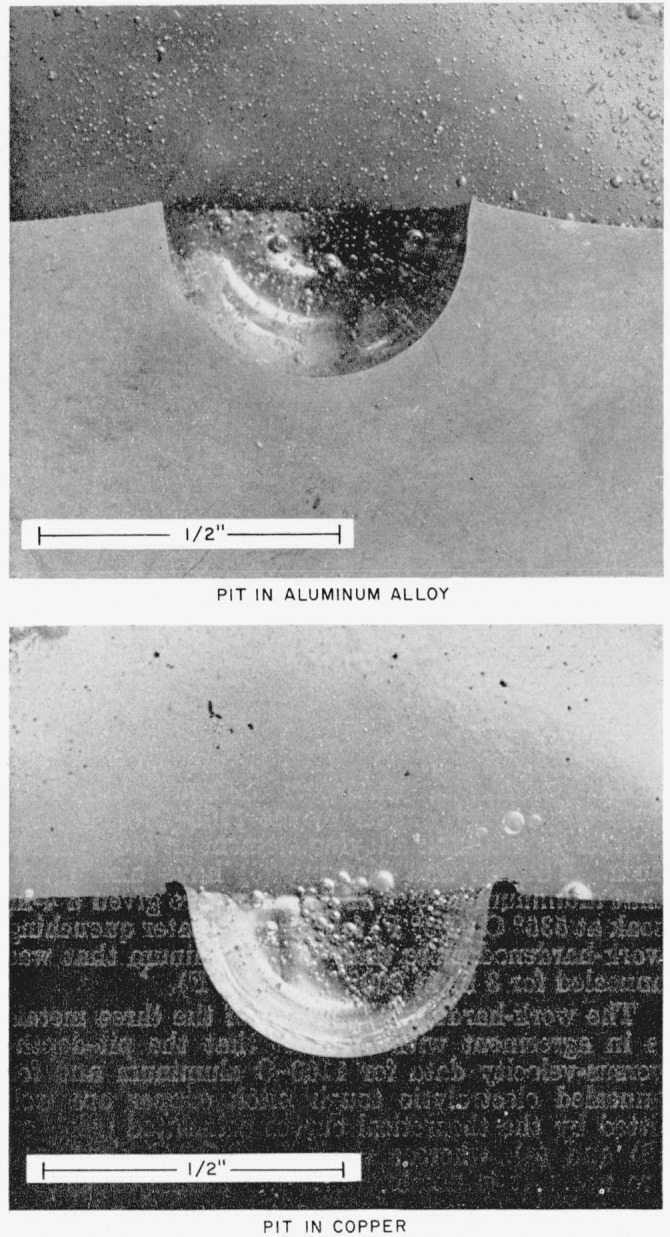
Cross sections of pits produced by collision of a 1.270-cm (½-in.) steel sphere with annealed electrolytic tough pitch copper and with 2024–O aluminum.

Cross-sectional cuts of the pits produced by these spheres were mounted in epoxy plastic in the NBS Mechanical Metallurgy Section (see fig. 8). They were given a high polish and examined with a microscope for evidence of crack formation. No evidence of crack formation was found. The cross section of the pit in 2024–O aluminum was then etched in an effort to accentuate any cracks if they existed, but no cracks were found. Crack formation in the target is, therefore, not the cause of the divergence of the 2024–O aluminum experimental pit-depth-versus-velocity data from the theoretical curves.

It was then thought that the compressional wave caused by the collision may have reflected as a tension wave from the free reverse surface of the 2024–O aluminum, target plate and may have returned to the collision surface with the effect of filling in the pits. This possibility is in agreement with the fact that attenuation of sound is greater in 1100–O aluminum and in annealed copper than it is in 2024–O aluminum.

To test this possibility a 1.59-cm-(⅝-in.-) thick plate of 1100 aluminum was cut to fit one of the 2.5-cm (1-in.-) thick target plates of 2024–O aluminum and was annealed under the same conditions as those that were used for the 1100–O aluminum targets. The contact surfaces between the two plates were machine ground and polished until, when they were pressed together, one plate was able to lift the other. They were tightly clamped together and 11 test firings were made against the combination target plate. The reverse (1100–O aluminum) face of the combination target plate was maintained as a free surface during the firings. Steel spheres 0.7938 cm ( 516 in.) in diameter were used for the shots.

It was hoped that if the compressional waves produced by the collisions had been returning to the collision surface as tension waves, they would now be prevented from doing this by attenuation on transit through the 1100–O aluminum. However, when the measured pit depths were plotted against the impingement velocity, it was found that the points were in complete agreement with those obtained without the 1100–O aluminum backing plate. The pit-depth-versus-velocity data are given in table 10. Apparently the return of the reflected tension wave to the collision surface is unable to explain the divergence of the 2024–O aluminum pit-depth-versus-velocity data from the theoretical curves. It is possible, however, that the degree of contact that was attained between the 2024–O aluminum target plate and 1100–O aluminum backing plate may not have been sufficient to ensure transmission of the elastic waves.

**Figure 9:**
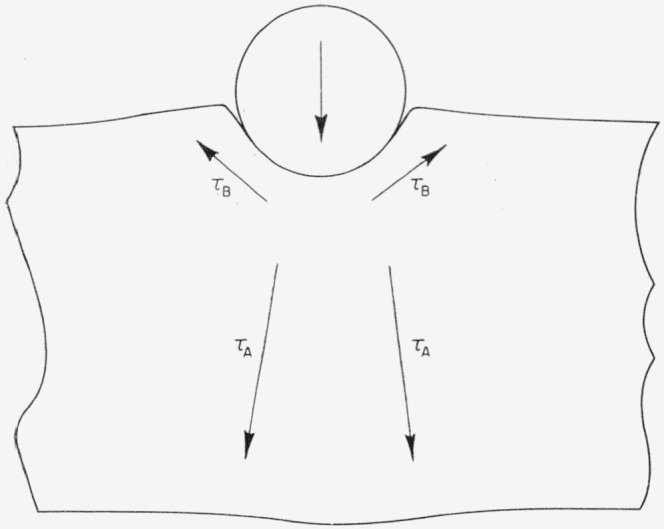
Shear stresses produced in a metal plate by an impinging steel sphere.

The explanation was finally sought in the work-hardening properties of the three target metals. When a rigid sphere impinges against a metal target plate, shear stresses exist around the cylinder of target metal that is set in motion as a result of the collision, as indicated by the arrows marked *τ_A_* in figure 9. Shear stresses also exist in the target metal around the point of impingement as indicated by the arrows marked *τ_B_* in figure 9. It seems reasonable that, if the metal does not work-harden readily, or if the *τ_B_*-shear stress is small, most of the plastic flow that takes place will occur as a result of the shear stress *τ_A_* that exists below the collision area. If, however, the metal work-hardens extensively while this process is initiated, then flow as a result of the shear stress *τ_A_* will be inhibited. In this case flow will occur as a result of the shear stress *τ_B_*, if the *τ_B_*-shear stress is appreciable, and the surface of the metal will be raised in a ring around the mouth of the pit that forms as a result of the collision.

It can be seen by laying a straight edge across the mouths of the crater cross sections shown in figure 8 that there is a considerable elevation of the target metal around the crater in the case of the 2024–O aluminum. Visual inspection of the target plates revealed that there was some rising of metal around the craters, especially in the case of the largest sphere size, for each of the target metals used. It appeared, however, to have occurred to a somewhat greater degree in the case of the 2024–O aluminum than in the case of the 1100–O aluminum or of the annealed electrolytic tough pitch copper.

Equations (1), (2), (3), and (4) were developed on the assumption that the principal movement that occurs as a result of a liquid-drop or of a steel-sphere collision with a metal target plate is that of the core of metal under the collision area [1]. If any other flow process (such as that resulting from the shear stress *τ_B_*) becomes appreciable in a specific target metal, these equations cannot be expected to apply to pits formed in that metal.

**Figure 10:**
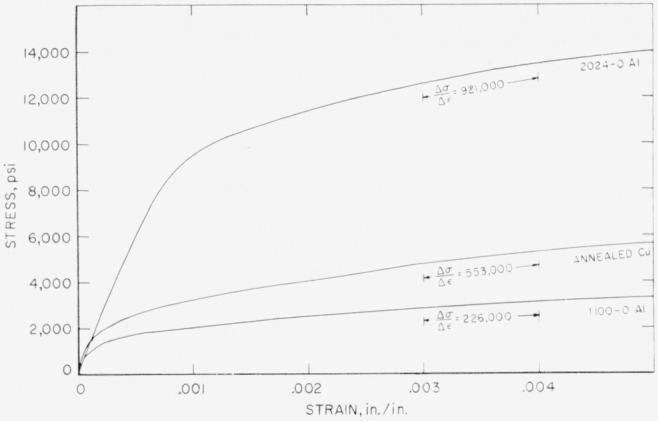
Stress-strain curves for 1100–O aluminum, 2024–O aluminum, and annealed electrolytic tough pitch copper.

The tensile stress-strain curves (fig. 10) provide a means of comparing the work-hardening properties of the 1100–O aluminum, annealed electrolytic tough pitch copper, and 2024–O aluminum used in the collision experiments. Work-hardening of metals may be evaluated by the tangent modulus [4]. The tangent moduli for the three metals in the range of strain from 0.003 to 0.004 are 1.56×10^10^ d/cm^2^ (226,000 psi), 3.81×10^10^ d/cm^2^ (553,000 psi), and 6.35×10^10^ d/cm^2^ (921,000 psi), respectively. These data suggest that the 2024–O aluminum work-hardens much more than does the annealed electrolytic tough pitch copper or the 1100–O aluminum. Copper is the main alloying element in 2024 aluminum. Thomas and Nutting [5] have also found that aluminum-copper alloys that were given a 4-hr soak at 535° C (995° F) followed by water quenching work-hardened more than pure aluminum that was annealed for 3 hr at 600° C (1,112° F).

The work-hardening behavior of the three metals is in agreement with the fact that the pit-depth-versus-velocity data for 1100–O aluminum and for annealed electrolytic tough pitch copper are well fitted by the theoretical curves calculated using eq (3) and (4), whereas those for 2024–O aluminum are not. It is possible that the plastic yield that occurs in 1100–O aluminum and in annealed electrolytic tough pitch copper as a result of impingement of steel spheres may be caused almost entirely by the shear stress *τ_A_*,whereas the plastic yield that occurs in 2024–O aluminum as a result of impingement of steel spheres may be caused both by the shear stress *τ_A_* and the shear stress *τ_B_*.

For mercury-drop and waterdrop impingement the pit-depth-versus-velocity data for 2024–O aluminum were well fitted by the theoretical curves calculated with use of eqs (1) and (2) (see fig. 2 and 4). This is not a contradiction because it is likely that the *τ_B_*-shear stress is smaller for the case of projectiles that flow daring and as a result of the collision than for the case of projectiles that do not flow.

More data, using steel-sphere projectiles and target metals that have different work-hardening properties, are needed to prove whether or not this explanation is correct.

### 4.4. Collisions of Steel Spheres With Target Plates of 2024–O Aluminum That Were Backed With a Heavy Steel Supporting Block

It has been pointed out that eqs (1), (2), (3), and (4) only apply to the case in which the target plate has edge support during the firings; the reverse side of the target plate must be a free surface. This is because the model on which eqs (1), (2), (3), and (4) are based involves the movement of the core of target material under the collision area with respect to the remainder of the target plate [1]. If the target plate is backed by a heavy metal plate or block, the reverse face of the target plate is not a free surface, the core of material under the collision area is not free to move with respect to the remainder of the target plate, and eqs (1), (2), (3), and (4) do not apply.

**Figure 11:**
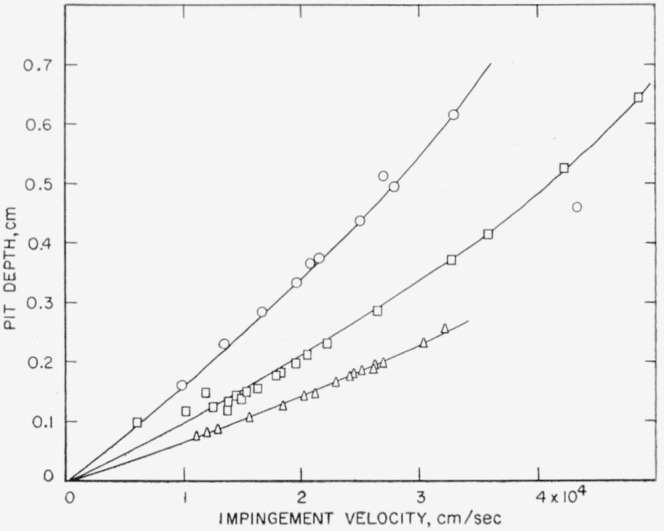
Best-fit curves for collisions of steel spheres against 2024–O aluminum targets backed with a heavy steel supporting block. ◬, observed depth produced by collision with 0.5556-cm ( 732-in.) spheres; ⊡, observed depth produced by collision with 0.7938-cm ( 516-in.) spheres; ⊙, observed depth produced by collision with 1.270-cm (½-in.) spheres.

Pit-depth-versus-velocity data were obtained for collisions of steel spheres of three sizes against 2.5-cm (1-in.-) thick target plates of 2024–O aluminum backed with a 12.7-by-15.2-cm (5-by-6-in.) steel supporting block 7.6-cm (3-in.) thick. These data are of no value as far as substantiation of eqs (1), (2), (3), and (4) is concerned. They are presented to show how the pit-depth-versus-velocity curve differs for the two modes of support of the target plate during the firings. These data are listed in table 11 and are plotted in figure 11 where best-fit curves have been drawn through the data for each size of steel sphere used. It can be seen from figures 7 and 11 that when the reverse side of the target plate is a free surface, the pit-depth-versus-velocity curve is a straight line, but that when the reverse side of the target plate is not a free surface (use of a backing plate or block), the pit-depth-versus-velocity curve is not a straight line.

For the case that the reverse face of the target plate is a free surface, the projectile is stopped by resistance to the movement of the core of metal under the contact area. For the case that the reverse face of the target plate is not a free surface, the projectile is stopped by resistance to extrusion of metal around the crater.

## 5. Liquid-Against-Liquid Collisions

Very little study has been made of collisions that occur when liquid drops collide with a target liquid. It was postulated by Opik [6] nearly 25 years ago, later by Pack and Evans [7], and recently by others [8] that at extremely high impingement velocities solid targets and projectiles will behave as though they were liquids. Öpik stated, “The ‘aerodynamic’ pressure at the penetration of a meteor into rock is of the order of 10^7^–10^8^ atmospheres, or more than 1,000 times the plastic limit of steel; no doubt all solid materials under such pressures must behave like liquids; thus the problem of meteor impact is the case of the impact of a liquid drop of given density *δ* into a liquid medium of density *ρ*.”

It has been found that when solid-sphere projectiles made of the soft ductile metals are fired at sufficiently high impingement velocities against targets of the same metal, they flow like liquids during and as a result of the collision. Pit depth for such solid-against-solid collisions has been found [1] to be given by eqs (1) and (2) which apply to collisions of liquid drops against metal plates.

If the impingement velocity is increased further, it is reasonable to suppose that the behavior predicted by Öpik [6] will eventually be found; the target as well as the projectile will liquefy during the collision. For such collisions that occur at meteor velocities Öpik [6] found that the impingement velocity has only a small effect upon the depth of penetration. If this is the case, the pit-depth-versus-velocity curve for such collisions should run *almost* parallel to the velocity axis. The penetration formula developed by Pack and Evans [7] has no velocity dependence.

**Figure 12:**
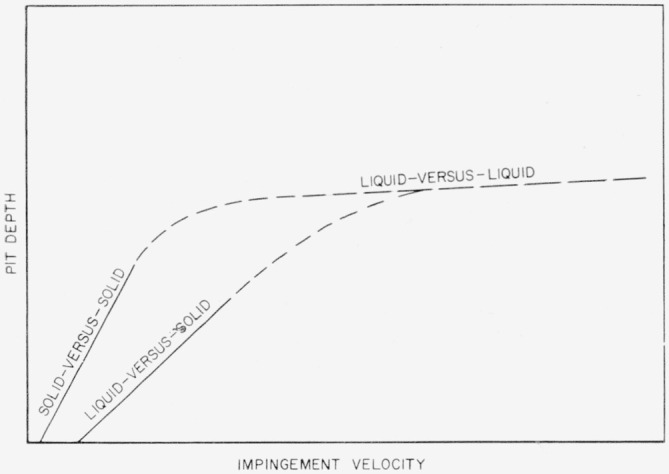
Schematic relation between various types of projectile-target collisions.

It appears that the straight-line, low-velocity, liquid-against-solid, and solid-against-solid pit-depth-versus-velocity curves for collisions of liquid drops and rigid steel spheres with metal plates must approach the high-speed liquid-against-liquid curve in some way when very high impingement velocities are reached. This is represented schematically in figure 12 where dashed lines have been used to indicate projected types of behavior.

Equations (1), (2), (3), and (4) will not apply either in the transition regions or in the velocity range of high-speed liquid-against-liquid collisions. An analysis of liquid-against-liquid collisions is in progress.

## Figures and Tables

**Table 1:** Static tensile properties of the metals

Metal	Yield strength		Tensile strength		Elongation

	10^8^ *d/cm*^2^	*psi*	10^8^ *d/cm*^2^	*psi*	%
1100–O aluminum	1.83	2,650	7.83	11,350	41.0
1100–O aluminum	1.79	2,600	7.65	11,100	41.5
2024–O aluminum	8.69	12,600	19.6	28,400	17.5
2024–O aluminum	8.76	12,700	20.0	29,000	17.5
2024–O aluminum	8.69	12,600	20.7	30,000	18.0
2024–O aluminum	8.69	12,600	20.9	30,300	18.5
Annealed copper	2.90	4,200	21.2	30,800	50.0
Annealed copper	2.96	4,300	21.4	31,000	49.0
Annealed copper	2.55	3,700	21.5	31,200	54.0
Annealed copper	2.55	3,700	21.4	31,100	53.0

**Table 2:** Constants of the materials of projectiles and targets

Constant		Projectile			Target		

		Water	Mercury	Steel	1100–O Aluminum	2024–O Aluminum	Copper electrolytic
Sound speed, *c*	cm/sec	a1.497×10^5^	a1.451×10^5^	b5.786×10^5^	b6.318×10^5^	b6.370×10^5^	b4.691×10^5^
Density, *ρ*	g/cm^3^	c0.99707	c13.546	d7.859	e2.713	e2.768	c8.92
Acoustic impedance^f^, *z*	g/cm^2^ sec	0.1493×10^6^	1.966×10^6^	4.547×10^6^	1.714×10^6^	1.763×10^6^	4.184×10^6^
Dynamic yield strength, *E*′	dynes/cm^2^	………	………	………	g7.239×10^8^	g2.350×10^9^	h2.394×10^9^

a Data from Bergmann [3].

b Measured in NBS Sound Section by C. E. Tschiegg.

c Data from Handbook of Chemistry and Physics.

d Data from Metals Handbook.

e Data from Aluminum Co. of America.

f Acoustic impedance is the product of sound speed and density, *z* = *cρ*.

g Dynamic yield strengths of the aluminum alloys were calculated from estimates of Whiffin, communicated by letter, for the ratio of the dynamic to the static yield strength.

h Dynamic yield strength of copper is that given by Whiffin [2].

**Table 3:** Experimental data^a^ for collisions of mercury drops of two sizes with annealed electrolytic tough pitch copper

0.1-cm drops			0.2-cm drops		

Collision velocity	Drop diameter	Pit depth	Collision velocity	Drop diameter	Pit depth

10^4^ *cm/sec*	*cm*	*cm*	10^4^ *cm/sec*	*cm*	*cm*
1.965	0.0946	0.009_4_	1.773	0.2027	0.029_2_
1.965	.0913	.010_2_	2.402	.2002	.047_8_
1.965	.1005	.014_0_	2.542	.2064	.066_0_
1.965	.0966	.016_2_	2.716	.1989	.059_7_
1.978	.1041	.016_3_	2.779	.1953	.058_7_
2.432	.0976	.021_6_	2.841	.2030	.064_0_
2.829	.1037	.029_5_	2.850	.2113	.074_7_
3.246	.1054	.039_1_	2.865	.2012	.065_5_
3.706	.1102	.044_4_	2.865	.2012	.067_8_
3.761	.0981	.043_7_	2.890	.2004	.065_0_
3.944	.1070	.051_3_	2.984	.1991	.077_0_
4.401	.1045	.058_7_	3.225	.2032	.082_3_
4.724	.0991	.061_5_	3.310	.1889	.076_7_
5.038	.0951	.059_9_	3.313	.1951	.076_6_
5.371	.0890	.064_5_	3.438	.2006	.073_7_
5.779	.0956	.074_2_	3.575	.1972	.094_7_
6.126	.1045	.083_6_	3.663	.2032	.131_8_
6.486	.0951	.084_6_	3.697	.1997	.083_6_
6.772	.1041	.097_3_	3.986	.2011	.123_2_
7.090	.0991	.093_0_	4.084	.2006	.107_3_
7.388	.1010	. 093_2_	4.096	.2004	.110_7_
7.526	.0991	.097_0_	4.322	.2048	.122_7_
8.074	.0935	.097_0_	4.401	.2003	.123_2_
8.409	.1065	.116_8_	4.450	.2025	.123_4_
8.708	.1019	.113_8_	4.862	.1990	.135_4_
9.832	.1028	.138_9_	5.919	.2099	.191_4_
10.50	.1036	.130_3_	6.069	.2046	.179_2_
			6.315	.2018	.177_5_
			6.410	.1995	.169_9_
			6.461	.1990	.199_9_

a See footnote 2.

**Table 4:** Experimental data^a^ for collisions of mercury drops of two sizes with 2024–O aluminum

0.1-cm drops			0.2-cm drops		

Collision velocity	Drop diameter	Pit depth	Collision velocity	Drop diameter	Pit depth

10^4^ *cm/sec*	*cm*	*cm*	10^4^ *cm/sec*	*cm*	*cm*
1.906	0.1032	0.080_0_	1.759	0.19514	0.012_4_
2.454	.0956	.020_1_	2.316	.19175	.027_2_
2.816	.0940	.0234	2.55_4_	.1998	.038_4_
2.890	.1032	.025_6_	2.682	.20122	.048_4_
3.413	.0976	.035_3_	2.789	.1969	.049_0_
3.627	.0996	.044_7_	3.048	.1984	.055_9_
3.797	.1037	.0490	3.200	.2017	.071_1_
3.822	.0996	.046_1_	3.377	.19672	.085_6_
4.322	.1037	.061_5_	3.596	.20330	.094_4_
4.669	.1009	.067_6_	4.008	.2047	.264_2_
4.745	.0946	.059_4_	4.084	.2031	.109_5_
4.995	.1041	.076_7_	4.517	.2010	.259_1_
5.145	.1000	.077_2_	5.489	.20572	.241_6_
5.517	.1058	.093_7_	5.727	.2000	.206_5_
5.752	.1014	.095_5_	6.834	.1982	.264_7_
6.004	.0971	.099_1_			
6.066	.0961	.096_8_			
6.251	.1032	.112_5_			
6.736	.1028	.130_8_			
7.047	.0971	.124_1_			
7.047	.0971	.149_9_			
7.525	.1032	.013_3_			
8.019	.1028	.146_6_			
8.071	.1037	.159_8_			
8.074	.1014	.148_8_			
8.525	.1023	.166_5_			
8.772	.1045	.176_3_			
9.195	.0946	.162_8_			
9.229	.0966	.163_6_			
9.623	.1019	.182_9_			
10.06	.0956	.162_6_			

a See footnote 2.

**Table 5:** Experimental data^a^ for collisions of 0.2-cm waterdrops with annealed electrolytic tough pitch copper

Collision velocity	Pit depth

10^4^ *cm/sec*	*cm*
4.593	0.003_0_
4.983	.005_1_
5.297	.009_4_
5.486	.003_6_
6.111	.010_4_
6.584	.009_9_
6.660	.006_1_
7.041	.013_0_
7.041	.019_3_
8.839	.017_0_

a See footnote 2.

**Table 6:** Experimental data^a^ for collisions of 0.2-cm waterdrops with 2024–O aluminum

Collision velocity	Pit depth

10^4^ *cm/sec*	*cm*
4.581	0.002_3_
4.581	.002_5_
4.852	.005_3_
5.456	.004_6_
6.370	.010_7_
6.584	.011_7_
7.224	.014_7_
7.498	.020_6_

a See footnote 2.

**Table 7:** Experimental data^a^ for collisions of steel spheres of three sizes with 1100–O aluminum

Sphere diameter 0.5556 cm (7/32 in.)		Sphere diameter 0.7938 cm (5/16 in.)		Sphere diameter 1.270 cm (1/2 in.)	

Velocity	Pit depth	Velocity	Pit depth	Velocity	Pit depth

10^4^ *cm/sec*	*cm*	10^4^ *cm/sec*	*cm*	10^4^ *cm/sec*	*cm*
0.9784	0.10_0_	0.7163	0.11_0_	0.887	0.22_4_
1.018	.10_4_	.9723	.14_6_	1.055	.27_2_
1.049	.10_7_	1.265	.19_2_	1.295	.32_9_
1.411	.14_8_	1.414	.21_2_	1.558	.37_5_
1.457	.15_1_	1.490	.22_4_	1.609	.39_2_
1.704	.18_0_	1.506	.23_4_	1.759	.44_8_
1.871	.19_9_	1.530	.23_4_	1.807	.46_6_
2.140	.22_9_	1.646	.25_3_	2.009	.52_6_
2.146	.23_3_	1.661	.25_6_	2.082	.55_6_
2.576	.29_1_	1.783	.27_6_	2.121	.60_8_
2.865	.34_4_	1.789	.27_5_	2.128	.55_4_
3.103	.37_3_	1.862	.29_7_	2.414	.65_6_
		1.993	.31_7_		
		2.137	.34_1_		
		2.423	.37_8_		

a These data were obtained at the U.S. Naval Research Laboratory, Washington, D.C.

**Table 8:** Experimental data^a^ for collisions of steel spheres of three sizes with annealed electrolytic tough pitch copper

Sphere diameter 0.5556 cm ( 732 in.)		Sphere diameter 0.7938 cm ( 516 in.)		Sphere diameter 1.270 cm (½ in.)	

Velocity	Pit depth	Velocity	Pit depth	Velocity	Pit depth

10^4^ *cm/sec*	*cm*	10^4^ *cm/sec*	cm	10^4^ *cm/sec*	*cm*
1.100	0.08_7_	0.7803	0.09_0_	0.4084	0.08_3_
1.372	.10_3_	.9388	.10_9_	.4389	.09_2_
1.500	.15_4_	1.119	.12_4_	.9601	.18_4_
1.878	.13_8_	1.283	.14_5_	1.353	.24_0_
1.981	.14_5_	1.588	.17_3_	1.411	.24_9_
2.121	.15_6_	1.740	.18_8_	1.426	.25_0_
2.124	.15_9_	2.280	.24_9_	1.451	.30_9_
2.158	.16_7_	2.377	.26_2_	1.868	.30_1_
2.225	.16_6_	2.457	.27_4_	1.966	.35_4_
2.234	.16_6_			2.036	.36_9_
2.256	.17_0_			2.060	.37_7_
2.271	.17_2_			2.134	.42_5_
2.316	.17_6_			2.204	.39_5_
2.646	.20_2_			2.216	.40_0_
2.685	.20_6_			2.694	.57_3_
2.960	.23_3_			2.704	.38_8_
3.560	.28_0_			3.066	.58_0_
				3.109	.49_5_
				3.225	.62_2_
				3.432	.63_8_

a These data were obtained at the U.S. Naval Research Laboratory, Washington, D.C.

**Table 9:** Experimental data^a^ for collisions of steel spheres of three sizes with 2024–O aluminum

Sphere diameter 0.5556 cm ( 732 in.)		Sphere diameter 0.7938 cm ( 516 in.)		Sphere diameter 1.270 cm (½ in.)	

Velocity	Pit depth	Velocity	Pit depth	Velocity	Pit depth

10^4^ *cm/sec*	*cm*	10^4^ *cm/sec*	*cm*	10^4^ *cm/sec*	*cm*
1.113	0.07_9_	0.7894	0.08_0_	0.5578	0.19_7_
1.451	.10_3_	1.067	.10_7_	.5822	.17_2_
1.542	.10_9_	1.305	.14_9_	1.207	.19_7_
1.981	.13_5_	1.698	.17_3_	1.326	.26_8_
2.173	.156	1.771	.18_0_	1.460	.24_8_
2.274	.18_8_	1.832	.18_5_	2.039	.34_5_
2.368	.16_9_	2.856	.349	2.722	.48_2_
2.448	.17_8_			2.780	.49_3_
2.701	.19_7_			2.786	.49_1_
				3.301	.62_2_
				3.484	.36_0_
				3.548	.62_2_
				3.658	.60_6_

a These data were obtained at the U.S. Naval Research Laboratory, Washington, D.C.

**Table 10:** Experimental data^a^ for collisions of 516-in. steel spheres with a 2024–O aluminum and 1100–O aluminum combination target

Collision velocity	Pit depth

10^4^ *cm/sec*	*cm*
0.9601	0.09_6_
1.201	.12_0_
1.478	.14_9_
1.640	.10_6_
1.890	.19_3_
2.103	.22_2_
2.603	.28_0_
2.734	.29_7_
2.841	.29_4_
3.203	.36_6_
3.755	.45_3_

a These data were obtained at the U.S. Naval Research Laboratory, Washington, D.C.

**Table 11:** Experimental data^a^ for collisions of steel spheres of three sizes with 2024–O aluminum backed with a steel supporting block

Sphere diameter 0.5556 cm ( 732 in.)		Sphere diameter 0.7938 cm ( 516 in.)		Sphere diameter 1.270 cm (½ in.)	

Velocity	Pit depth	Velocity	Pit depth	Velocity	Pit depth

10^4^ *cm/sec*	*cm*	10^4^ *cm/sec*	*cm*	10^4^ *cm/sec*	*cm*
1.106	0.74_4_	0.6035	0.09_9_	0.9753	0.16_0_
1.192	.08_0_	1.012	.11_5_	.9845	.16_2_
1.295	.08_7_	1.186	.14_7_	1.341	.23_0_
1.548	.10_6_	1.247	.12_1_	1.664	.28_3_
1.844	.12_7_	1.356	.11_7_	1.966	.33_2_
2.027	.14_1_	1.369	.13_3_	2.094	.37_0_
2.115	.14_6_	1.384	.13_5_	2.143	.37_4_
2.298	.16_5_	1.384	.13_6_	2.146	.37_5_
2.408	.17_4_	1.417	.13_3_	2.502	.43_8_
2.438	.17_9_	1.436	.14_0_	2.697	.51_2_
2. 502	.18_2_	1.481	.13_9_	2.780	.49_5_
2.609	.18_7_	1.503	.14_9_	3.283	.61_8_
2.615	.19_3_	1.622	.15_7_	4.331	.46_1_
2.615	.18_8_	1.786	.17_8_		
2.688	.19_8_	1.823	.18_4_		
3.036	.23_1_	1.939	.19_7_		
3.216	.25_2_	2.057	.21_1_		
		2.216	.23_0_		
		2.640	.28_7_		
		3.255	.37_2_		
		3.572	.41_6_		
		4.209	.52_9_		
		4.846	.64_6_		

a These data were obtained at the U.S. Naval Research Laboratory, Washington, D.C.

## Footnotes

1 Figures in brackets indicate the literature references at the end of this paper.

2 These data were obtained at Convair, Division of General Dynamics Corp., in San Diego, Calif. See reference [1].
